# Selections

**Published:** 1891-12

**Authors:** 


### Selections.

### WHY SYPHILIS IS NOT ABORTED BY THE EARLY DESTRUCTION OR EXCISION OF ITS INITIAL LESION.

By R. W. TAYLOR, M. D.,
Clinical Professor of Venereal Diseases at the College of Physicians and Surgeons, New York.

From a very early date the idea of aborting syphilis by various means was entertained by surgeons, and this idea has prevailed until the present day. Ten or twelve years ago excision of chancre, as a prophylactic measure, was largely practiced, and the communications are very numerous bearing upon the subject. The consensus of opinion among advanced syphilographers of to-day is that the operation is utterly futile. I have held for many years that the operation (useful in some cases as a therapeutic procedure) is utterly valueless as a means of destroying syphilis, and I have been searching for a long time the reason why.

The following personal case I will give with considerable detail, for upon it largely hangs the conclusions reached in this essay. On January 3, 1890, a gentleman came to me wishing to make an appointment for a date upon which I could circumcise him. At that time I made a preliminary examination of his penis, and on the free border of the prepuce I found a small, brownish-red, not excoriated, papule, no larger than the head of a small tack. It struck me instantly that he had a hard chancre, for I had many times seen almost precisely such an insignificant lesion develop into a true syphilitic sclerosis. The patient was positive that when he took his bath on the previous morning there was no spot on his penis, and he thought there had been none in the evening. So circumstances seemed to warrant the view that it began in the night.

Upon questioning the gentleman I obtained the following facts: He lived in the suburbs of a large Southern city, and, as a rule, when he went to town he cohabited with a woman in some first-class house. He had been at his home, and had for a period of three months prior to December 20, 1889, been perfectly continent. On that date he went to the city, transacted business during the day, and passed the night in the embraces of a lady of the town, whom he had been assured was perfectly clean and healthy. He had no further sexual intercourse until the time of his first visit to me, just fourteen days after this last coitus. At the consultation I explained my suspicions, thus fortified by a very clear and conclusive history, and urged the operation, thinking in my own mind that if ever there was a favorable opportunity for testing the value of excision of chancre as a prophylactic for syphilis, this was the case. It so happened that business of importance delayed the operation until January 7th, four days after the appearance of the chancre, and eighteen days after the inoculation of the syphilitic infection. At this time no perceptible enlargement of the inguinal ganglia could be felt, a symptom looked for and also not found four days previously. The prepuce was removed, and, owing to its marked redundancy fully one and one-half inch of tissue was ablated. Healing took place promptly in a week, and no traces of induration were to be found in the cicatrix. Plenty of tissue remained to allow of full erections. The patient was watchful of himself and constantly came for inspection. On January 15th, I thought I detected slight enlargement of the inguinal ganglia ; but on the twentieth of that month the adenopathy was very evident. From that date it increased until it became typical in all particulars. On February 21st, a typical roseola appeared, which had been watched for daily during the preceding fortnight. At this time the ganglia in the neck and at the elbows were perceptibly enlarged and typically syphilitic. Prior to the evolution of the secondary period of syphilis I found very minute but hard cords running up the dorsum of the penis. The nature of the excised papule was therefore proved beyond doubt, and I congratulated myself that

I had placed in a proper fluid for preservation and examination that redundant but precious prepuce.

These studies, therefore, go to show that in the very first days of syphilitic infection, as shown by the chancre after the first period of incubation, the poison is deeply rooted beneath the initial lesion, and that it extends far beyond it; that it is in a most active state, and, running along the course of the vessels, it soon infects all the parts beyond, even to the root of the penis. These studies seem to warrant the conclusion that the virus is not localized to its point of entry and that it does not shut itself in by throwing out a dense wall of circumvallation, which later on disappears and allows of the exudation of the morbid products of the heretofore supposed closed-in morbid focus. Clinical and pathological facts go to show that the infective process occurs very rapidly.

Ricord, in his later years, has said that he considered the destruction of the infecting chancre as absolutely useless (in a prophylatic sense), no matter how early it is done. That it is certain, even before its appearance, that syphilis exists, and that even if the entire penis should be amputated before the chancre showed itself, syphilis would follow nevertheless. This certainly is rather a broad statement, but in a measure it is warranted by the facts which have just been developed.

In this essay I have sought to show why syphilis is not aborted by excision of its initial lesion, with a liberal slice of the surrounding parts. The reason, succinctly stated, is, that (contrary to the present view) the syphilitic infective process is from the very start a quite rapid one. That the poison strikes directly for the blood vessels and causing there its peculiar changes, runs along them with astonishing rapidity. Thus it gains a foothold in parts beyond the reach of the knife, the caustics, or electrolysis. In fact, the tissues of the whole penis in very early syphilis are, we may say, honeycombed by these infected vessels. These observations just presented, backed by the evidence of the failures in chancre excision, go to show that beyond the chancre there is sufficient syphilitic poison to infect the whole economy, and that the initial lesion, though the visible and exuberant evidence of syphilitic infection, may be removed without in any way altering or modifying the course of the disease. It is rather too early to inquire into the *modus operandi* of the maturing syphilitic infection, but it seems probable that this vessel cell-growth goes on and on until the whole economy is involved, and that then the explosion occurs which we call the evolution of the secondary period of the disease.—*Medical Record.*

